# Tailoring light delivery for optogenetics by modal demultiplexing in tapered optical fibers

**DOI:** 10.1038/s41598-018-22790-z

**Published:** 2018-03-13

**Authors:** Marco Pisanello, Filippo Pisano, Leonardo Sileo, Emanuela Maglie, Elisa Bellistri, Barbara Spagnolo, Gil Mandelbaum, Bernardo L. Sabatini, Massimo De Vittorio, Ferruccio Pisanello

**Affiliations:** 1 Istituto Italiano di Tecnologia (IIT), Center for Biomolecular Nanotechnologies, 73010 Arnesano (LE), Italy; 2 Dipartimento di Ingegneria dell’Innovazione, Università del Salento, Lecce, Italy; 3 Department of Neurobiology, Howard Hughes Medical Institute, Harvard Medical School, Boston, 02115 MA USA

## Abstract

Optogenetic control of neural activity in deep brain regions ideally requires precise and flexible light delivery with non-invasive devices. To this end, Tapered Optical Fibers (TFs) represent a versatile tool that can deliver light over either large brain volumes or spatially confined sub-regions, while being sensibly smaller than flat-cleaved optical fibers. In this work, we report on the possibility of further extending light emission length along the taper in the range 0.4 mm-3.0 mm by increasing the numerical aperture of the TFs to NA = 0.66. We investigated the dependence between the input angle of light (θ_in_) and the output position along the taper, finding that for θ_in_ > 10° this relationship is linear. This mode-division demultiplexing property of the taper was confirmed with a ray tracing model and characterized for 473 nm and 561 nm light in quasi-transparent solution and in brain slices, with the two wavelengths used to illuminate simultaneously two different regions of the brain using only one waveguide. The results presented in this manuscript can guide neuroscientists to design their optogenetic experiments on the base of this mode-division demultiplexing approach, providing a tool that potentially allow for dynamic targeting of regions with diverse extension, from the mouse VTA up to the macaque visual cortex.

## Introduction

Over the past decade, optogenetic stimulation has had a remarkable impact on neuroscience research as it permits millisecond precision manipulation of genetically-targeted neural populations^1–3^. However, harnessing the full potential of the optogenetic approach requires light to be delivered to the opsin-transfected neuron under tight spatiotemporal control. This task is even more arduous when targeting brain regions beyond 1 mm depth, as common light-delivery methods (cleaved fiber optics and two-photon excitation) fail in penetrating such depths or are highly invasive. Driven by the experimental need for novel approaches, research in innovative technologies for deep brain light delivery has produced several promising solutions such as multi-dimensional wave-guides or high-density µLED probes^4–20^. In a recent work^21^ we demonstrated a simple and cost-effective device based on a thin Tapered Optical Fiber (TF) that can perform both homogeneous light delivery and dynamically-controlled spatially-restricted illumination over brain regions extending up to ~1.8 mm in the dorso-ventral direction. This was done by exploiting TFs light-emission properties that are determined by the subset of guided modes that propagates in the taper^22^. Experiments from two independent research groups have shown in the last year the great advantages of TFs for *in vivo* control of neural activity in different animal models. For example, wide-volume illumination was obtained in the motor cortex and in the striatum of both free-moving and head restrained mice^21^, as well as in the Frontal Eye Field of non-human primates^23^. TFs also allowed for site selective light delivery in the striatum to control specific locomotion behavior, on the base of different modal subsets injected into the fiber to illuminate ventral or dorsal striatum using only one implant^21^.

Building on this background, here we analyze the use of high-NA fibers to obtain wide volume or spatially-selective light-delivery over ~3 mm, a light emission length that is suitable to cover the extent of brain regions of several animal models, from mice to non-human primates. We demonstrate that a linear relationship exists between the position of the emission region along the taper and the input angle at which light is injected into the fiber. In addition, we characterize mode-division demultiplexing from 0.66NA TF at 473 nm and 561 nm in fluorescent solution and in mouse brain slices. Assessing TFs light-delivery performances at longer wavelengths is indeed a crucial aspect for using this technology with a broad set of opsins^24–26^. The versatility of TFs as customizable light-delivery tools may be seen as a promising resource for neuroscientists seeking to obtain bi-directional optogenetic modulation of neural activity in shallow or deep brain regions, with the results reported in this paper giving access to a set of quantitative characterization useful for experimental design with a wider range of opsins in multiple regions of the brain.

## Theoretical Background

**Figure 1:**
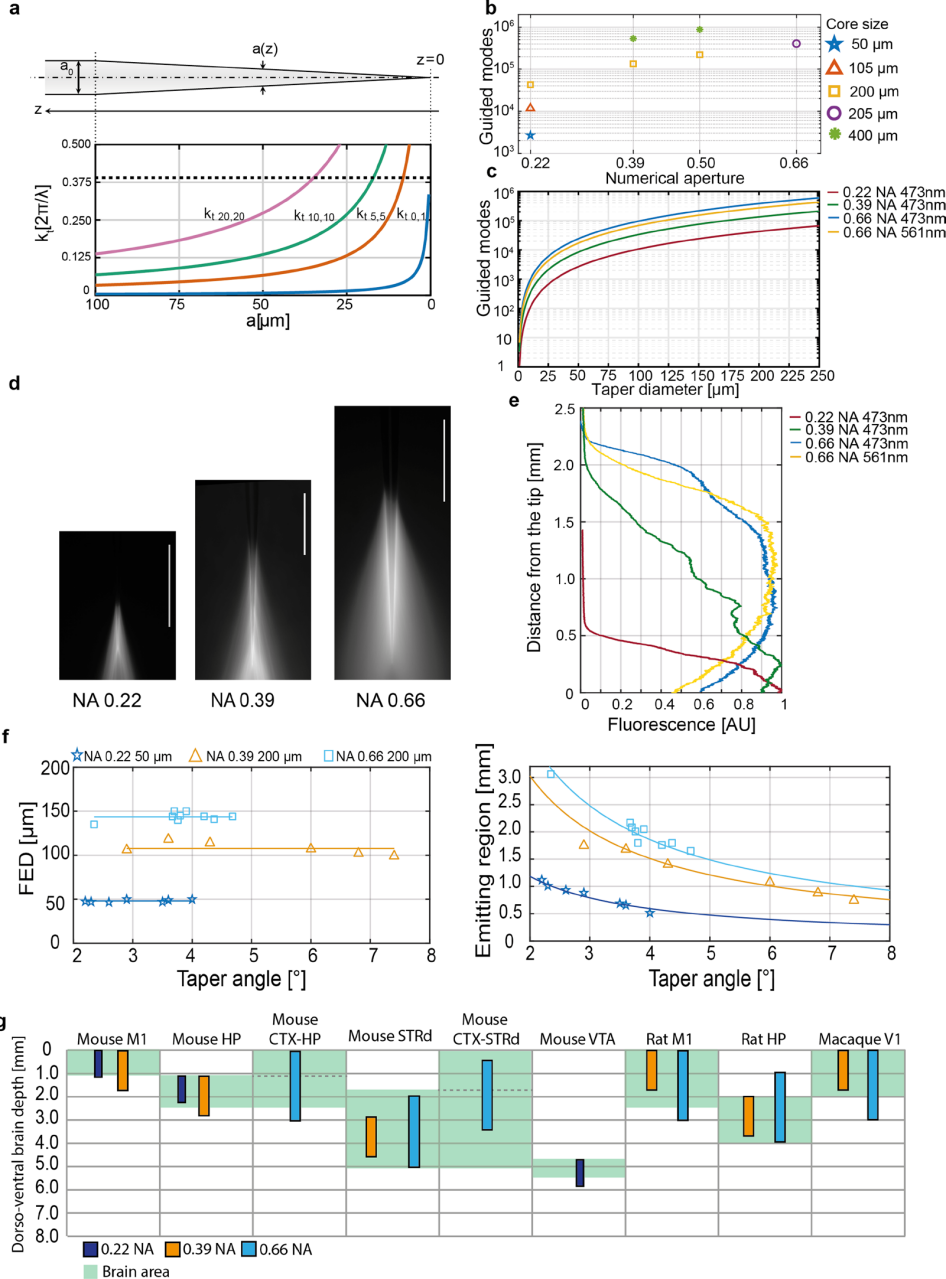
Tailoring TF devices emission lengths. (**a**) *(top)* Schematic representation of a tapered optical fiber*. (bottom)* Evolution of the transversal propagation constant of four different guided modes as a function of the taper diameter. **(b)** Number of modes sustained by fiber optics with increasing core size and NA. (**c**) Number of guided modes at increasing diameters of the tapered section for fibers with 50 µm core/0.22 NA at 473 nm (red curve), 200 µm core/0.39 NA at 473 nm (green curve), and 200 µm core/0.66 NA at 473 nm (blue curve) and 561 nm (yellow curve). (**d**) Fluorescence image of light emission for a fiber with 50 µm core/0.22 NA *(left)*, 200 µm core/0.39 NA *(center)*, and 200 µm core/0.66 NA *(right)*, ~3.7° taper angle, injected with 473 nm light; scale bars are 1 mm. **(e)** Normalized intensity profile measured along the taper surface for the three fibers in panel (d) injected with 473 nm light; the yellow curve represents the emission profile of a NA = 0.66 TF injected with 561 nm light. **(f)** First Emission Diameters (*left*) and Emission Lengths (*right*) for different fiber types with increasing taper angle. (**g**) Diagram of dorso-ventral depth of brain regions targetable with optogenetic stimulation in mouse, rat and macaque. The shaded areas in the background represent the dorso-ventral extension of each region as obtained from on-line brain atlas. Colored bars display the maximum emission length provided by TFs with NA = 0.22 (dark blue), NA = 0.39 (orange), and NA = 0.66 (pale blue). M1, primary motor cortex; HP hippocampus; CTX, cortex; STRd, dorsal striatum; VTA, ventral Tegmental Area; V1, primary visual cortex. For panels d–g, data pertaining 0.22 NA and 0.39 NA fibers were reused from Pisanello *et al*.^21^.

When injecting light in the whole fiber numerical aperture (NA) with a Gaussian beam, light-delivery devices based on TFs, schematically displayed in Fig. 1a, can illuminate large brain volumes by emitting radiation from a long segment of the taper surface^21,23^. This occurs because of a gradual loss of light along the taper generated by the progressive narrowing of the waveguide diameter *a(z)*. Given a fiber with initial diameter *a*_0_, the transversal propagation constants k_t_ of the guided modes increases from the initial value *k*_*t*_(*a*_*0*_) following the relation^27^

1 $${k}_{t}(a)\approx \frac{{a}_{0}}{a}{k}_{t}({a}_{0}).$$

The behavior of some *k*_t_ values as a function of *a* is shown in Fig. 1a as a reference for the reader. Since the taper can support light propagation only for modes with^27^

2 $${k}_{t}(a) < {k}_{t,\max }={k}_{0}{\rm{NA}}=\frac{2\pi }{\lambda }\text{NA},$$

where λ is the propagating wavelength, each mode that does not fulfill this constraint is out-coupled. If the radiation injected into the tapered region of the waveguide is composed by guided modes with *k*_*t*_ in the interval [0, *k*_*t,max*_], the condition in equation 2 will be gradually broken along the taper. This is shown in Fig. 1a where transverse propagation constants (colored lines) overtake the *k*_*t,max*_ threshold (dashed line). As a consequence, higher order modes with higher transversal propagation constants tend to be out-coupled at larger taper diameters. Low order modes with low *k*_*t*_ are instead out-coupled close to the taper tip. It follows that an optical fiber supporting a larger *k*_*t,max*_ and sustaining a higher number of modes is expected to produce tapered sections with a longer region able to optically interface with the environment. Following Snyder *et al*.^27^, the total number of guided modes (*M(z)*) for a section of diameter *a(z)* can be approximated as

3 $$M(z)\approx \frac{1}{2}{[\frac{\pi }{\lambda }a(z){\rm{NA}}]}^{2}.$$

As a direct consequence, fibers with higher core size and higher NA can sustain a larger number of guided modes (Fig. 1b) and the number of guided modes diminishes when approaching the fiber tip (Fig. 1c). These two parameters are reported in Fig. 1b and c, respectively, as a support for the following description.

## Results

### Effects of NA and maximum transverse propagation constant on the emission properties of tapered optical fibers

Building on the well-known properties described in Section 2, we considered three optical fibers that support an increasing number of modes distributed over widening intervals of transverse constants [0, k_t,max_] with similar taper angles (ψ~3.7°). Namely, we investigated fibers with NA = 0.66 with *a*_0_ = 200 µm and compared them with previous results for fibers with NA = 0.22 and *a*_0_ = 50 µm and with NA = 0.39 and *a*_0_ = 200 µm^21^.

The extent of the taper segment emitting light (hereafter referred to as “emitting length”, EL) was measured as the distance from the tip at which the detected fluorescence decreases to half of the intensity at the fiber tip (see emission profiles displayed in Fig. 1d,e and detailed definition in Methods). For the same taper angle (ψ~3.7°), TFs with higher NA are optically active over a longer taper region (Fig. 1e), as relation 2 starts to be broken farther from the taper tip. In detail, 0.22 NA fibers show EL~484 µm, for 0.39 NA TFs EL~1220 µm, and EL~2038 µm for 0.66 NA. Data pertaining 0.22 NA and 0.39 NA fibers were reused from Pisanello *et al*.^21^, with the goal to compare higher NA TFs performance with known TFs. It is interesting to notice that NA = 0.66 TF have a shorter EL (1874 µm) when injected with 561 nm light (Fig. 1e, yellow curve), as expected due to the lower number of guided modes (and lower *k*_*t,max*_) at red-shifted wavelengths (see Fig. 1c).

Although the number of guided modes decreases as soon as the waveguide narrows (Fig. 1c) and relation (2) is broken just after the taper entrance for modes with high *k*_*t*_ (Fig. 1a), light emission starts to be appreciable in the fluorescence measurements only at a First Emission Diameter (FED) that depends on fiber NA and waveguide size (Fig. 1f). This can be ascribed to the fact that high order modes have higher propagation losses with respect to low order modes and they are excited with lower power efficiency by a Gaussian beam focused on the fiber core^27,28^. As shown in Fig. 1f, fibers with large core size and large NA (e.g. supporting a larger number of modes and a higher *k*_*t,max*_) lead to higher values of the FED, which do not depend on the taper angle ψ. For the fibers investigated here, this leads to the observation that the higher the NA, the higher the ratio between the FED and the fiber diameter at the taper entrance: 55/125 = 0.44 for NA = 0.22, 126/225 = 0.56 for NA = 0.39 and 154/230 = 0.67 for NA = 0.66. At the same time, we observed a diminished FED (130 µm) for NA = 0.66 TFs when injected with 561 nm light. This is consistent with equations 1–2, as a longer wavelength implies a smaller k_t,max_ that in turn translates to a smaller FED. In Fig. 1f, data pertaining 0.22 NA and 0.39 NA fibers were reused from Pisanello *et al*.^21^, with the goal to compare higher NA TFs performance with known TFs. Therefore, 0.66NA fibers are expected to support the longest ELs.

Given the independence of FED on ψ, EL is expected to depend on the taper angle for a linear taper profile following the relation

4 $${\rm{EL}}=\frac{\alpha \cdot {\rm{FED}}}{2\cdot \,\tan (\psi /2)},$$

as shown by the good agreement with experimental data displayed Fig. 1f. The scale factor α was defined as the ratio between the EL and the FED distance from the taper tip for ψ = 3.7°, and was similar across NAs (α_NA=0.66_ = 0.8652, α_NA=0.39_ = 0.8956 and α_NA=0.22_ = 0.8848). The fiber allowing for the longest emission for the same ψ is the one that distributes the widest range of *k*_*t*_ on the widest number of guided modes, i.e. the 0.66NA fiber. In particular, increasing numerical aperture to 0.66, allowed reaching emission lengths of ~3 mm, against the ~1.8 mm obtained previously^21^. In light of these results, the FED can be identified as a chief design parameter for TFs, taking into account the effects on taper emission of several constitutive parameters of the fiber: the number of guided modes that propagates in the taper and of their *k*_*t*_ values in relation with *k*_*t,max*_.

The relation linking TFs emission length to NA and taper angle (equation 4) allows choosing the correct device to match the size of targeted structures in the living brain. As schematically shown in Fig. 1g, probes with NA = 0.22 and limited core size (*a*_*0*_ = 50 µm) are suited to target regions in mouse cortex or deep nuclei such as the ventral tegmental area (VTA). While 0.22 NA/50 µm core fibers can illuminate a relevant portion of the mouse hippocampus, devices with larger NA and core, such as 0.39 TFs with 200 µm core, are more appropriate for this task as they can be engineered to illuminate up to 1.8 mm in the dorso-ventral axis. These TFs can be applied to target both dorsal and ventral, for instance. However, this emission length cannot cover both a cortical-striatal projecting region and the dorsal ventral axis of mouse striatum. In addition it cannot cover the full extent of rat motor cortex (M1) or the macaque visual cortex (V1) depth. The introduction of 0.66NA TFs is therefore essential to efficiently deliver light in several functional regions, as shown in Fig. 1g. These TFs can also potentially cover the full dorso-ventral extension of the above-mentioned regions and might be exploited to simultaneously reach cortical and subcortical regions in the mouse and the rat (Figs 1g, 4). The shaded areas in the background represent the dorso-ventral extension of each region as obtained from on-line brain atlases^29–31^.

### Linear control of TFs emission regions

As previously demonstrated, the light emitting portion of TFs can be dynamically controlled by remotely adjusting the light input angle^21^. In fact, coupling light into TFs with an angle-selective launching system selects well-defined subsets of bounded modes that propagate into the optical fiber, which are then out-coupled at different taper sections^21^. In the following the relationship between the input angle and the out-coupling position along the taper is quantitatively estimated.

**Figure 2:**
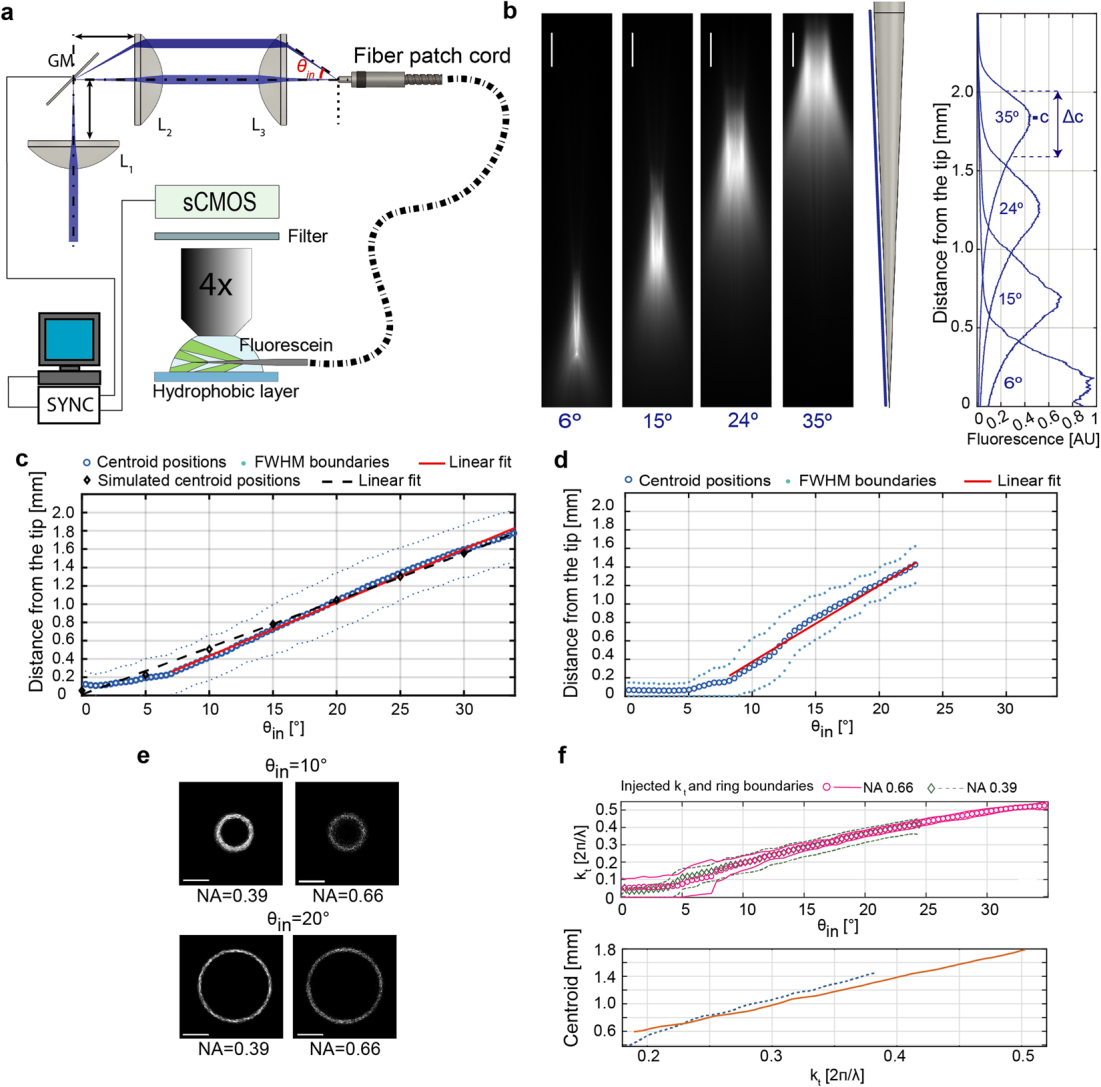
Spatially selective light delivery using high-NA TF. (**a**) Diagram of the optical setup used for mode division demultiplexing (L1, L2, L3 lenses; GM galvanometric mirror). Laser light enters the fiber patch cord at an angle and is out-coupled from the taper surface immersed in fluorescein. A sCMOS camera acquires fluorescence images of the light emission. The GM motion is synchronized with the camera acquisition via custom software (see Methods). (**b**) *Left*, emission images for a NA = 0.66, 200 µm core size fiber with increasing input angle. Scale bars are 250 µm. *Center*, diagram of the tapered section. The blue line indicates the profile along which the intensity was measured. *Right*, intensity profiles measured at different input angles with respect to the distance from the tip. Four selected regions are selected by varying the input angle. (**c**) Position of the intensity centroid of the emission region (blue circles) and positions of the FWHM values of the emission region (pale blue dots) with respect to the input angle for a NA = 0.66 TF. The red line indicates a linear fit performed on the centroid position. Ray tracing simulations results are displayed as black diamonds, with a linear fit shown as a black dashed line. (**d**) As in panel (**c**) for a NA = 0.39 TFs. (**e**) Far field images of the patch cord injected with θ = 10°, 20° at 473 nm. Scale bars are 0.15∙2π/λ. (**f**) *Top*, centroid and boundaries of the injected k_t_-subset versus input angle. *Bottom*, centroid position versus the injected k_t_ for 0.66 NA, orange line, and 0.39 NA, dashed blue line.

To this purpose the light redirection system displayed in Fig. 2a was implemented: a lens L1 focuses a Gaussian laser beam onto the rotation axis of a galvanometer mirror (GM). The beam is deflected by the GM of an angle θ_GM_, collimated by lens L2 and focused onto the fiber by lens L3 at an angle θ_in_. The taper was submerged in a PBS:fluorescein bath to analyse the light emission geometry through an epi-fluorescence microscope as a function of θ_in_. The GM deflection was synchronized with the acquisition camera (see Methods) to speed up the acquisition of closely spaced emission profiles (~0.5° step in input angle). 0.39 NA and 0.66 NA TFs with 200 µm core diameter were tested, both with ψ~3.7°. As discussed in the previous paragraph, 0.66 NA fibers sustain a high number of guided modes (~4.0 × 10^5^) distributed over a large ensemble of transverse propagation constants (*k*_*t,max*_ = 8.7 × 10^−3^ nm^−1^ for λ = 473 nm). Representative images of light emission for four different θ_in_ are displayed in Fig. 2b. As shown by the emission profiles in the proximity of the linear taper surface (Fig. 2b, right), it is possible to dynamically redirect light output over four different emission regions with equally spaced emission peaks. By extracting the centroid of the emission region (*c*) and the relative FWHM (*Δc*) from the emission profile at each input angle (definitions of *c* and *Δc* in Fig. 2b and in Methods), we found that *c* depends linearly on the input angle θ for θ > 10° (with a slope of ∼58 µm/°, fit RMSE of 11 µm) and can be dynamically moved along ~1.8 mm (Fig. 2c). Moreover, the FWHM was found to be approximately constant at *Δc* = 550 ± 5 µm for θ > 10°. This behaviour was confirmed by ray tracing simulations that, as displayed in Fig. 2c, highlight the linear relation between the position of the emission region and θ. Experimental and simulated data are in good agreement (Fig. 2c). For 0.39 NA fibers with the same taper angle, supporting a lower number of modes (~1.3 × 10^5^ at 473 nm) and a narrower range of *k*_*t*_ (*k*_*t,max*_ = 5.2 × 10^−3^ nm^−1^ for λ = 473 nm), *c* can be moved along ~1.4 mm, although the position of the centroid slightly deviates from a linear dependence (slope fit of ∼50 µm/°, fit RMSE of 76 µm), as shown in Fig. 2d.

Building on these measurements, we analyzed the relationships between guided modes and emission properties of TFs with different numerical apertures. To confirm that the observed difference between geometrically identical 0.39 NA and 0.66NA TFs is related to the different extent of *k*_*t*_ values sustained by the two fibers, we used the method proposed in ref.^22^ to measure the subsets of *k*_*t*_ injected into 0.66 NA and 0.39 NA TFs for each input angle. Briefly, we injected light on a patch cord fiber and then imaged far field of the fiber emission on a CCD camera (Supplementary Figure 1). As shown in Fig. 2e, this produced light rings whose diameter is proportional to the input angle and can be linked to the transverse propagation constant *k*_*t*_. In order to characterize the injection of modal subset with a *k*_*t*_*(θ)* ± Δ*k*_*t*_*(θ)* curve, we extracted the output ring diameter and its FWHM for each input angle (see Methods). The results of the measurement are reported in Fig. 2f (top). As expected, *k*_*t*_*(θ)* values for 0.39 and 0.66 NA fibers are almost overlapping for θ > 10°. For the same injected *k*_*t*_ value, emission from the 0.66 NA TF is closer to the fiber tip (Fig. 2f, bottom) due to the higher refractive index, and light emission can be moved on a wider taper extent by virtue of the higher *k*_*t,max*_ supported by the 0.66 NA TF.

**Figure 3:**
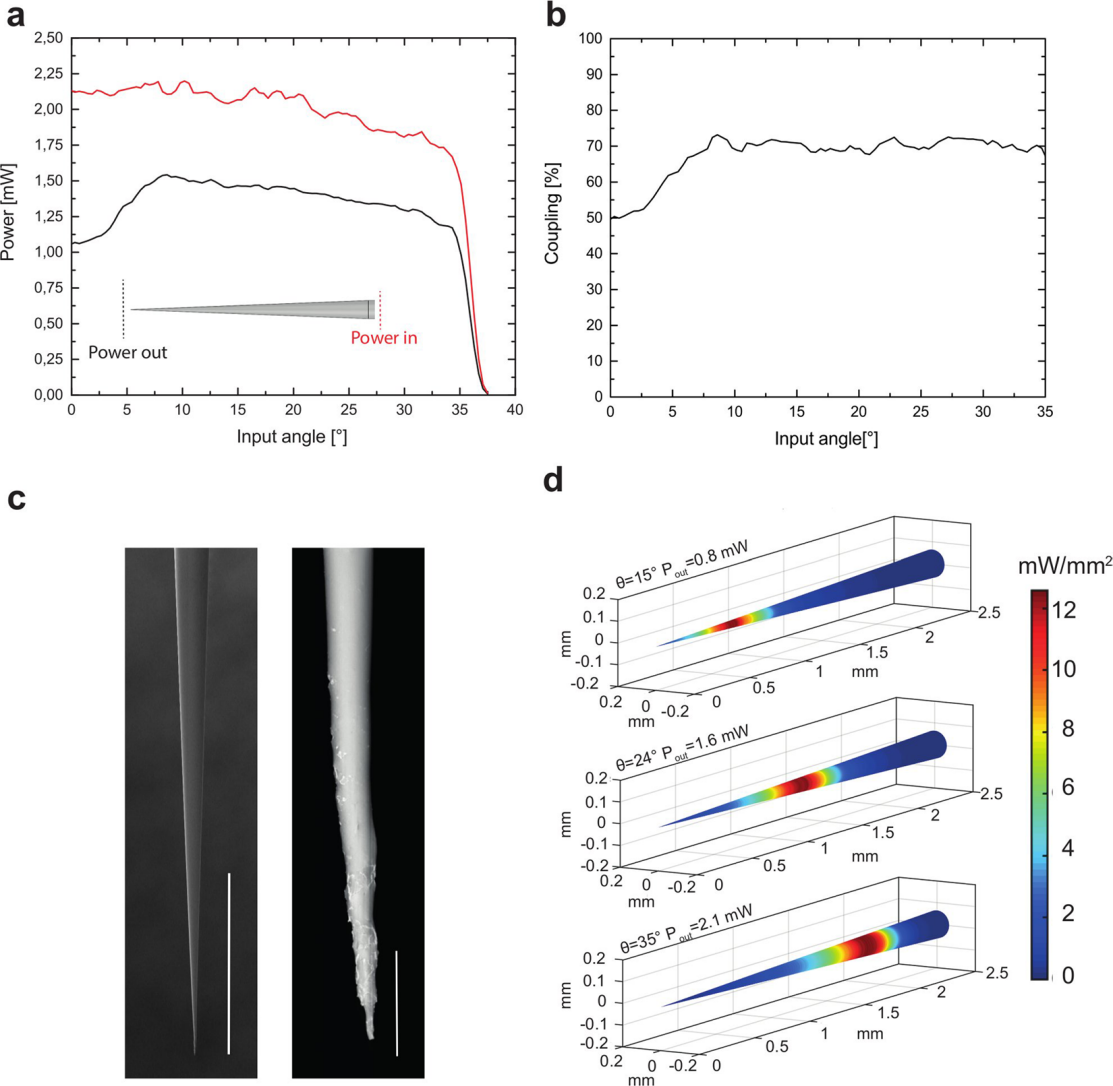
Characterization of light coupling efficiency and output power density for NA = 0.66 TFs. (**a**) Light coupling across the NA = 0.66 angular acceptance. The input power (red line) was measured at the entrance of the tapered region. The output power (black line) was measures in close proximity to the taper tip, as shown in the inset. A drop in power output was observed for low input angles. (**b**) Power coupling efficiency, calculated as the ratio between output and input power, versus light input angle. The coupling efficiency is approximately constant for θ_in_ > 10°. (**c**) Scanning electron microscope images of a NA 0.66 TF. While the left panel shows a homogeneous portion of the taper region, scale bar 400 µm, the close-up in the right panel shows degradation of the taper surface in proximity of the tip, scale bar is 20 µm. (**d**) The three panels show the distribution of power density on the taper surface when 12 mW/mm^2^ are emitted for three input angles, namely θ_in_ = 15°, 24°, 35°. As light is out-coupled from a wider region when the input angle increases, the input power has to be varied accordingly to maintain a constant output power density.

Another important aspect to evaluate the influence of injected *k*_*t*_ values on TFs light emission properties is the different attenuation of light injected at different θ_in_ as high order modes have higher attenuation constant than do low order modes^27,28^. This is visible in the measurements displayed in Fig. 3a (red curve): before entering the taper, light power slightly decreases as a function of θ_in_ and the same happens at the taper output (black curve). Interestingly, in the range of input angle for which *c* depends linearly on θ_in_ (i.e. θ_in_ > 10°), the coupling efficiency between the taper input and the taper output remains approximately constant, as shown in Fig. 3b. A drop in coupling efficiency was observed for low injection angles. This effect was explained by SEM inspection, which revealed lower surface quality at the very tip of the taper (Fig. 3c). On the basis of these considerations, the output power density distribution around the taper for different input angles was assessed from the measured emission profiles (see Methods for details on the performed calculations). Sample diagrams for three different θ_in_ in the linear range are shown in Fig. 3d. The average power density can be maintained constant over the different emission regions by slightly increasing the total output power as a function of θ_in_, compensating for the increase of the optically active surface of emitting regions at higher diameters. For the particular case shown in Fig. 3d, an increase of ~3 fold, roughly similar to the increase in optically active surface, holds the average power density constant (12 mW/mm^2^) for θ_in_ = 15°, 24°, 35°. This is well above the widely recognized threshold for obtaining optogenetic control of neural activity with ChR2 (1 mW/mm^2^)^26^ and obtained with relatively low input powers (ranging from 0.8 to 2.1 mW).

### Yellow and dual-wavelength light delivery with high-NA TFs in brain tissue

**Figure 4:**
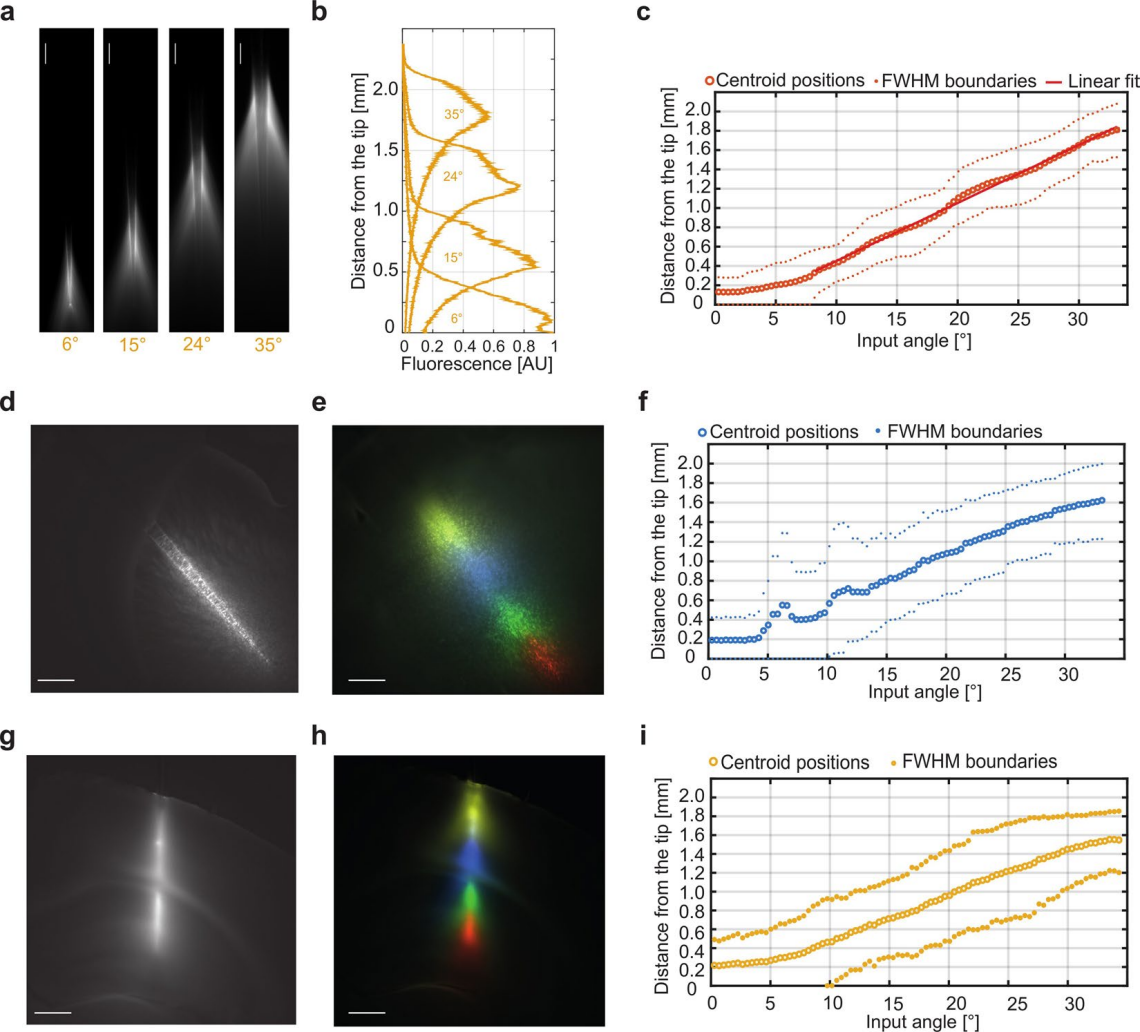
High NA TFs: light delivery at 561 nm and in brain tissue. (**a**) Spatially selective light delivery observed for a NA = 0.66, 200 µm core size fiber injected with 561 nm light and submerged in a PBS-eosin solution. Scale bars are 200 µm (**b**) Emission profiles measure along the taper surface for the input angles shown in panel (**a**). (**c**) Position of the intensity centroid of the emission region (orange circles) and positions of the FWHM values of the emission region (pale orange dots) with respect to the input angle. The red line indicates a linear fit on the centroid position. (**d**) Large volume illumination obtained by injecting 473 nm light over the fiber full NA once inserted in mouse striatum; scale bar is 500 µm. (**e**) False color overlay of adjacent regions illuminated with spatially selective light delivery at 473 nm, scale bar is 500 µm. (**f**) Position of the intensity centroid of the emission region (blue circles) and positions of the FWHM values of the emission region (pale blue dots) with respect to the input angle. (**g**) Large volume illumination obtained by injecting 561 nm light over the fiber full NA once inserted in mouse cortex and hippocampus; scale bar is 500 µm. (**h**) False color overlay of adjacent regions illuminated with spatially selective light delivery at 561 nm, scale bar is 500 µm. (**g**) Position of the intensity centroid of the emission region (yellow circles) and positions of the FWHM values of the emission region (yellow dots) with respect to the input angle.

New optogenetic actuators have been developed in the last years, with activation peaks spanning the visible spectrum^24–26^. In particular, optogenetic inhibition of neural activity has been demonstrated by delivering yellow light over a neural population transfected with inhibiting opsin probes, such as Halorhodopsin^26^. Moreover, a recent work used a TF-based device to achieve neuronal inactivation over a large volume in the non-human primate cortex by targeting a red light-sensitive halorhodopsin (Jaws) with 635 nm light^23^. Since both the number of guided modes and the width of the k_t_ values sustained by TFs decrease at higher wavelengths (M**~**4 × 10^5^, 3 × 10^5^, 2 × 10^5^ for 473, 561 nm and 635 nm respectively, see equations 2 and 3), it is important to assess TFs performances for site-selective optogenetic inhibition. Fluorescence emission profiles for angle-selective injection (Fig. 4a,b) were acquired in a water:eosin solution (see Methods) to test fiber response at 561 nm light. As observed for blue wavelengths, we found that the position of the emission region centroid moves linearly as a function of the input angle for θ_in_ > 10° (Fig. 4c) (fit slope ∼60 µm/° and RMSE 25 µm).

In order to verify that the emission features described so-far are not modified by tissue absorption and scattering, light delivery performances were tested in brain tissue by inserting 0.66 NA TFs in fixed mouse brain slices previously stained with SYBR green and MitoTracker deep red to test fibers properties at 473 nm and 561 nm, respectively. Figure 4d shows the full NA emission in the mouse striatum with 473 nm light exciting SYBR green. Spatially selective light delivery was characterized by stimulating fluorescence emission from the taper while remotely varying the input angle with a step of 0.5°. Figure 4e shows a false color overlay of the fluorescent emission excited over four adjacent brain regions with 473 nm light. Images 4d-e were acquired with a green fluorescence filter (525/50). As for the characterization in fluorescein, we used the emission profiles measured along the taper surface to extract centroid *c* and FWHM *Δc* of the emission region for each input angle. As shown in Fig. 4f, scattering from brain tissue slightly widens the distribution of the light emitted for a given modal subset, but the overall behavior of *c vs θ*_*in*_ is not affected, with some deviations from the linearity measured in fluorescein due to the non-homogeneity of tissue scattering. We then performed an analogous measurement by inserting a NA = 0.66 TF of similar taper angle in a brain slice stained with MitoTracker deep red. Figure 4g shows the full NA emission distributed between cortex and hippocampus with 561 nm light. Afterwards, we repeated the evaluation of site selective mode-division demultiplexing. Figure 4h shows a false color overlay of the fluorescent emission excited over four adjacent brain regions with 561 nm light. Images 4g-h were acquired with a red fluorescent filter (716/40). The position of the centroid c and FWHM *Δc* were extracted and reported in the graph in Fig. 4i. Also in this case, a wider *Δc* was observed due to tissue scattering, with an almost-preserved linear behavior as a function of *θ*_*in*_.

**Figure 5:**
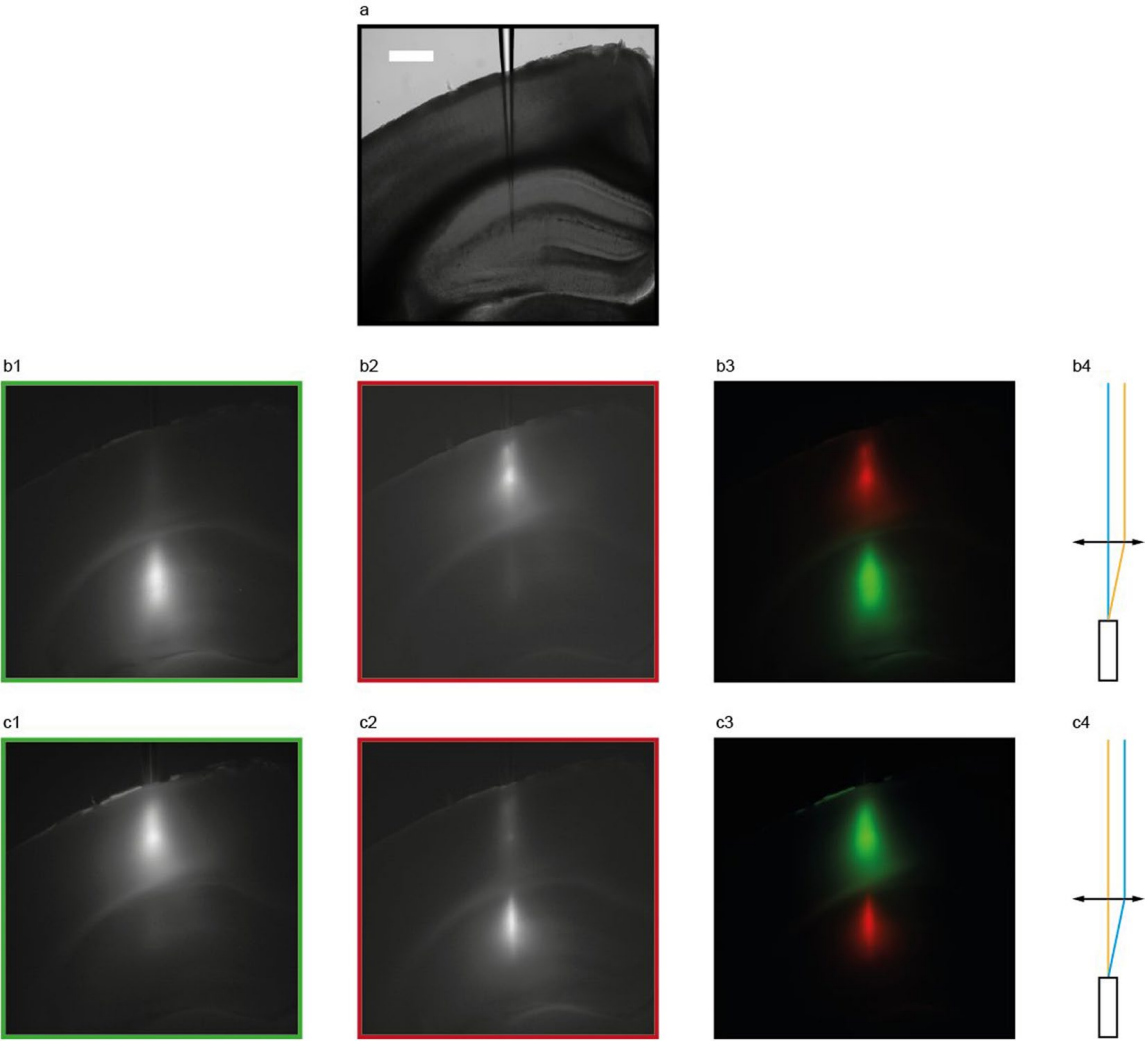
Simultaneous dual-wavelength mode-division demultiplexing in brain tissue with high NA TF. (**a**) Brightfield image of the location of a NA = 0.66 TF inserted in a mouse coronal brain slice with the tip reaching the hippocampus. Scale bar is 500 µm and is common to all micrographs in the figure. (**b**) Green (b1), red (b2) and overlay (b3) channels of simultaneous excitation of SYBR green fluorescence in the hippocampus and MitoTracker deep-red fluorescence in the cortex. Panel (b4) schematically shows the light injection configuration, with 473 nm entering at ∼4° and 561 nm entering at ∼20°. (**c**) Green (c1), red (c2) and overlay (c3) channels of simultaneous excitation of SYBR green fluorescence in the cortex and MitoTracker deep-red fluorescence in the hippocampus. Panel (c4) schematically shows the light injection configuration, with 473 nm entering at ~20° and 561 nm entering at ~5°.

Simultaneous mode-division demultiplexing at 473 nm and 561 nm was demonstrated by independently routing two laser beams into a TF inserted in a coronal mouse brain slice co-stained with SYBR-green and MitoTracker deep-red. Figure 5a shows a NA = 0.66 TF inserted in a coronal brain slice with the tip reaching the hippocampus. We simultaneously confined light emission from SYBR-green in the hippocampus and MitoTracker deep-red in the cortex (Fig. 5b), and then swapped the excitations (Fig. 5c). Red and green channels images were acquired by changing the fluorescence filters during simultaneous excitation at 473 nm and 561 nm (see Methods). Figures 5b3–c3 show overlays of the two channels, respectively acquired for each configuration, as schematically depicted in fig. 5b4–c4. Supplementary Figure 3 shows co-localized light stimulation at 473 nm and 561 nm both in the cortex (Supplementary Figure 3b) and the hippocampus (Supplementary Figure 3c). These measurements confirm that a single TF device can perform independent dual-wavelength light delivery over two functionally distinct brain regions.

## Discussion

Delivering light in a controlled fashion is required in order to fully exploit the potential of optogenetic techniques in controlling and monitoring neural circuits^32^. To this end, light delivery based on TFs offers an innovative tool to reach deep brain regions that are precluded to ordinary optogenetic stimulation protocols. By using 0.22, 0.39 and 0.66 TFs the possibility of tailoring emission length of TFs devices is extended to the interval ~0.4 mm to ~3 mm, with a simple relationship (equation 4) to define taper angle and fiber NA. This tuning range well matches with the extent of brain structures of different animals including mice, rats and non-human primates, as shown in Fig. 1g.

By injecting defined subsets of guided modes within specific k_t_ intervals, far field analysis allowed measurement of the direct relationship between the light output position along the taper and the injected k_t_ values. This revealed a linear dependence for input angles θ_in_ > 10° for both blue and yellow light. Within the linear region, the output power remains almost constant, while the emitted power density slightly decreases due to the increase of waveguide diameter when the emitting region moves farther from the taper tip.

TFs represent a unique approach to deliver light into the living brain with reduced invasiveness, as they allow for site-selective stimulation over wide volumes without the implantation of electric devices or of multiple waveguides. Although uncoated TFs cannot produce an illumination spot as tight as microfabricated TFs^22,33^ or cylindrical fibers^34^, they provide the user with dynamic re-configuration between wide-volume and site-selective light emission. Moreover, there is no need to determine the illumination area *a priori*, as light emission can be moved continuously across the entire optically active range^21^. Therefore, we view these approaches as complementary rather than exclusive.

All these features let us envision that the here-presented results can help neuroscientists in improving experimental protocols targeting sub-cortical regions of the mouse brain, functional structures of larger rodents and non-human primates, as well as analysis methods not compatible with conducting metals (such as opto-fMRI), placing TFs as a great complement to available methods to deliver light into brain.

## Materials and Methods

### Device structure and fabrication process

Tapered optical fibers with taper angles ranging from 2° to 8°, fabricated from fiber cords with NA = 0.22 core/cladding = 50/125 µm (Thorlabs FG050UGA), NA = 0.39 core/cladding = 200/225 µm (Thorlabs FT200UMT), NA = 0.66 core/cladding = 200/230 µm (Plexon PlexBright High Performace patch cable) were obtained from OptogeniX (www.optogenix.com). Tapers were fabricated by heat-and-pull method using a laser puller. Output power of the CO_2_ laser, scanning length over the fiber, and pulling force temporal pattern have been optimized to obtain the desired taper shapes. Details of the procedure are provided in previous works^21,35^. Fibers were connected to ceramic or metallic ferrules with a diameter of 1.25 mm. Supplementary Figure 4 shows cross section images of the tapered region for NA = 0.66 and NA 0.39 fibers. These were obtained by cutting and polishing the 0.39 and 0.66 TFs at diameters of ~85 µm and ~125 µm respectively with the aid of a 127 µm bare ferrule. Interestingly, while no sign of polymeric cladding was observed for NA = 0.39 fibers, the boro-silicate cladding in NA = 0.66 TFs can be distinguished. The ratio between the core and cladding diameter at the investigated section (110/125 = 0.88) and at the input facet of the fiber (200/230 = 0.87) is constant, suggesting that core and cladding reduce proportionally in the NA = 0.66 fibers used.

### Emission properties characterization

#### Data acquisition

The emission properties of the devices were characterized with two different light coupling systems: *(i)* injection of the whole numerical aperture accepted by the fiber and *(ii)* injection of a defined input angle *θ*_*in*_. Laser light was injected at 473 nm (Laser Quantum Ciel 473) and 561 nm (Coherent OBIS 561 nm LS). In both systems, the taper was coupled to a patch fiber with matching NA and core size by a ferrule to ferrule butt-coupling. In the full NA injection configuration of case (*i*) light was coupled to the fiber patch cord with an Olympus objective AMEP-4625 (focal length 4.5 mm, N.A. = 0.65), or with fiberports (Thorlabs PAF-SMA-5-A focal length 4.6 mm, N.A. = 0.47, PAF-SMA-7-A focal length 7.5 mm, N.A. = 0.29). To fill the entire clear aperture of the coupling lens, the laser beam was expanded by a proper factor through a beam expander realized with off-the-shelf lenses.

The angle-selective launch system of case *(ii)* was implemented using a galvanometric-mirror based scanning system. Two lenses L1 and L2 (Thorlabs LA1050-A with focal length *f*_1_ = 100 mm and AL50100-A with focal length *f*_2_ = 100 mm, respectively) relayed the laser beam and converted the angular deflection of the GM mirror (Sutter RESSCAN-GEN) into a displacement *t* perpendicular to the optical axis of the system. Lens L3 (Thorlabs AL4532-A with focal length *f*_3_^(c)^ = 32 mm and a 45 mm aperture) focused the light onto the patch fiber core. The actual input angle *θ* was measured by registering the displacement of the laser spot on a digital camera placed in front of the coupling lens for a known mirror deflection. The tapered fibers were immersed in a PBS:fluorescein or PBS:eosin solution to image light emission patterns with input wavelengths of 473 nm or 561 nm, respectively. Images were acquired using an upright epi-fluorescence upright microscope (Scientifica Slicescope) equipped with a 4 × objective (Olympus XLFLUOR4X/340 with immersion cap XL-CAP), fluorescence emission filters (525/50 for fluorescein emission and 605/70 for eosin emission) and a sCMOS camera (Hamamatsu ORCA-Flash4.0 V2). Optical output power was measured in air with a power meter (Thorlabs PM100USB with S120VC sensor head). Power coupling efficiency was measured as the ratio between taper and patch fiber optical power output.

Tapered fiber emission in brain slices was measured by inserting the light emitting region of the taper in coronal mouse brain slices fixed in PFA and stained with SYBR green (S9430, Sigma Aldrich) or both SYBR green and Mitotracker deep-red (M22426, Thermo Fisher Scientific). Excitation laser light at 473 nm and/or 561 nm was coupled with system (*i*) and (*ii*) into the tapered fiber. Detection of fluorescence was performed through an epi-fluorescence upright microscope (Scientifica Slicescope) equipped with a 4 × objective (Olympus XLFLUOR4X/340 with immersion cap XL-CAP) and a sCMOS camera (Hamamatsu ORCA-Flash4.0 V2). Simultaneous light injection at 473 nm and 561 nm was obtained by combining two independent scan path in front of lens L3 using a beam splitting cube mounted on a translation stage (Supplementary Figure 5). Images were acquired by subsequently filtering SYBR-green and MitoTracker deep-red emission with fluorescence filters (525/50 and 716/40 respectively). Supplementary Figure 6 shows a proposed setup to independently perform simultaneous mode division demultiplexing with three different wavelengths by combining three different galvanometric scanners by means of dichroic mirrors.

#### Data analysis

A set of intensity profiles was extracted from the fluorescence images collected with coupling scheme *(i)* and *(ii)*. Profiles were measured along a line drawn just outside the taper, parallel to the waveguide surface. Profiles given by full N.A. excitation were used to quantify both the First Emission Diameter (FED) and the total length of the emission region (Emission Length, EL). The FED was defined as the position at which the intensity recorded by the sCMOS sensor is half of the average intensity detected in the pixel with a recorded intensity exceeding 90% of the maximum recorded intensity (Supplementary Figure 2). The same intensity profiles were used to estimate the power density distribution along the taper when the coupling scheme *(ii)* was used. Assuming a rotational symmetry around the taper axis of the power density distribution *p*, a Matlab code has been developed to calculate *p* starting from the total output power and the intensity profile extracted from the image. Spatial selectivity in light out-coupling from the taper was quantified from the profiles measured with angle-selective excitation. The centroid and Full Width at Half Maximum (FWHM) positions of the emission region were measured for each input angle *θ* with respect to the fiber tip. Linear fits as function of the input angle *θ* were computed on the centroid position and both the FWHM boundaries positions. Data points for which the lower bound of the emission region was coincident with the fiber tip were excluded from the fit computation because the presence of the taper tip affects these measurements by breaking the shape symmetry.

### Injected *k*_*t*_ measurements

The value of the transversal propagation constant *k*_t_ of the light guided by the optical fiber as a function of the input angle was measured by imaging the farfield pattern of the light outcoupled by the facet of an optical fiber stub while changing *θ* at the input facet, as previously described^22,36^. In particular, Olympus objective AMEP-4625 (focal length 4.5 mm, N.A. = 0.65) was used to generate the farfield pattern, and a two-lens imaging system (realized with Thorlabs AC254-040-A-ML and AL50100-A) was used to magnify the pattern over the chip of the sCMOS sensor (Hamamatsu ORCA-Flash4.0 v2). The signal detected at distance *r* from the center of the chip is associated to *k*_t_ by the equation

5 $$r=\frac{4.5\,{\rm{mm}}\cdot 100.0\,{\rm{mm}}}{40.0\,{\rm{mm}}}\,\tan ({\sin }^{-1}(\frac{\lambda }{2\pi }{k}_{t})).$$

For each input angle, the k_t_ at which the maximum signal is detected and the half-prominence width of the peak were extracted from the disk- or ring-shaped images recorded by the sCMOS sensor.

#### Ray tracing simulations

Ray tracing simulations were performed with the commercial optical ray tracing software Zemax-OpticStudio (http://zemax.com). 0.66NA TFs were modeled as straight core/cladding nested cylinders followed by a conical taper section with 3.7° angle. Core/cladding refractive indexes were set as core = 1.63 and cladding = 1.49 as retrieved from ref.^37^. Tapered sections were modeled by nesting two cones with a common vertex. Core/cladding diameters were set as the manufacturer nominal values of 200/230 µm. The length of the core/cladding non-tapered segment was set to 50 mm. The source (5 M rays) was modeled as a circular homogeneous light distribution coupled in the fiber with an ideal paraxial lens. Different input angles were simulated by modulating the tilt of the source-lens system with respect to the fiber axis. The irradiance profiles of the emitted light were detected by placing a rectangular, pixelated detector (6000 × 41) in close proximity (~50 µm) of the taper surface.

### Data availability

Data are available from the corresponding author on reasonable request.

## Electronic supplementary material

Supplementary Figures
