# Supplementary material for: Tailoring light delivery for optogenetics by modal demultiplexing in tapered optical fibers
Source: Sci Rep. 2018 Mar 13;8:4467. doi: 10.1038/s41598-018-22790-z (PMC5849750; doi:10.1038/s41598-018-22790-z)
Supplement: Supplementary file 1 — Supplementary Figures [file 41598_2018_22790_MOESM1_ESM.doc]

**SUPPLEMENTARY INFORMATION**

**Tailoring light delivery for optogenetics by modal demultiplexing in tapered optical fibers**

Marco Pisanello^1,2,+^, Filippo Pisano^1,+^, Leonardo Sileo^1^, Emanuela Maglie^1,2^, Elisa Bellistri^1^, Barbara Spagnolo^1^, Gil Mandelbaum^3^, Bernardo L. Sabatini^3^, Massimo De Vittorio^1,2^, Ferruccio Pisanello^1,*^

^1^Istituto Italiano di Tecnologia (IIT), Center for Biomolecular Nanotechnologies, 73010 Arnesano (LE), Italy.

^2^Dipartimento di Ingegneria dell’Innovazione, Università del Salento, Lecce, Italy.

^3^Department of Neurobiology, Howard Hughes Medical Institute, Harvard Medical School, Boston, 02115 MA, U.S.A.

^*^ferruccio.pisanello@iit.it

^+^These authors contributed equally to this work.

**Supplementary figure 1**


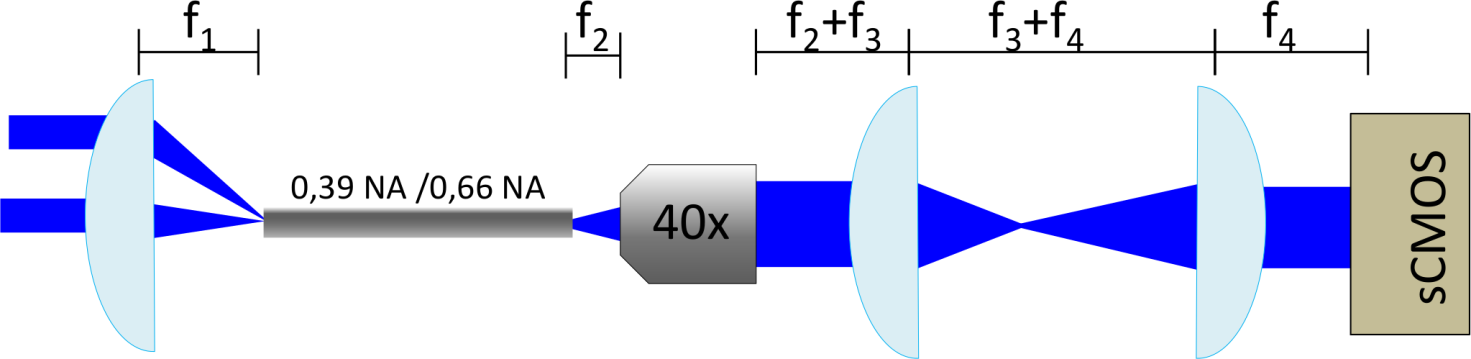


**Supplementary figure 1:** Optical system for far-field imaging of the fiber output. Light is injected into the fiber with an aspheric NA=0.61 lens (f1=32 mm). Light emitted from the fiber is collected with a 40x objective (f2=4.5 mm) and relayed on a sCMOS chip with an achromatic doublet, f3=40mm, and a 50 mm,f4=100 mm lens.

**Supplementary figure 2**


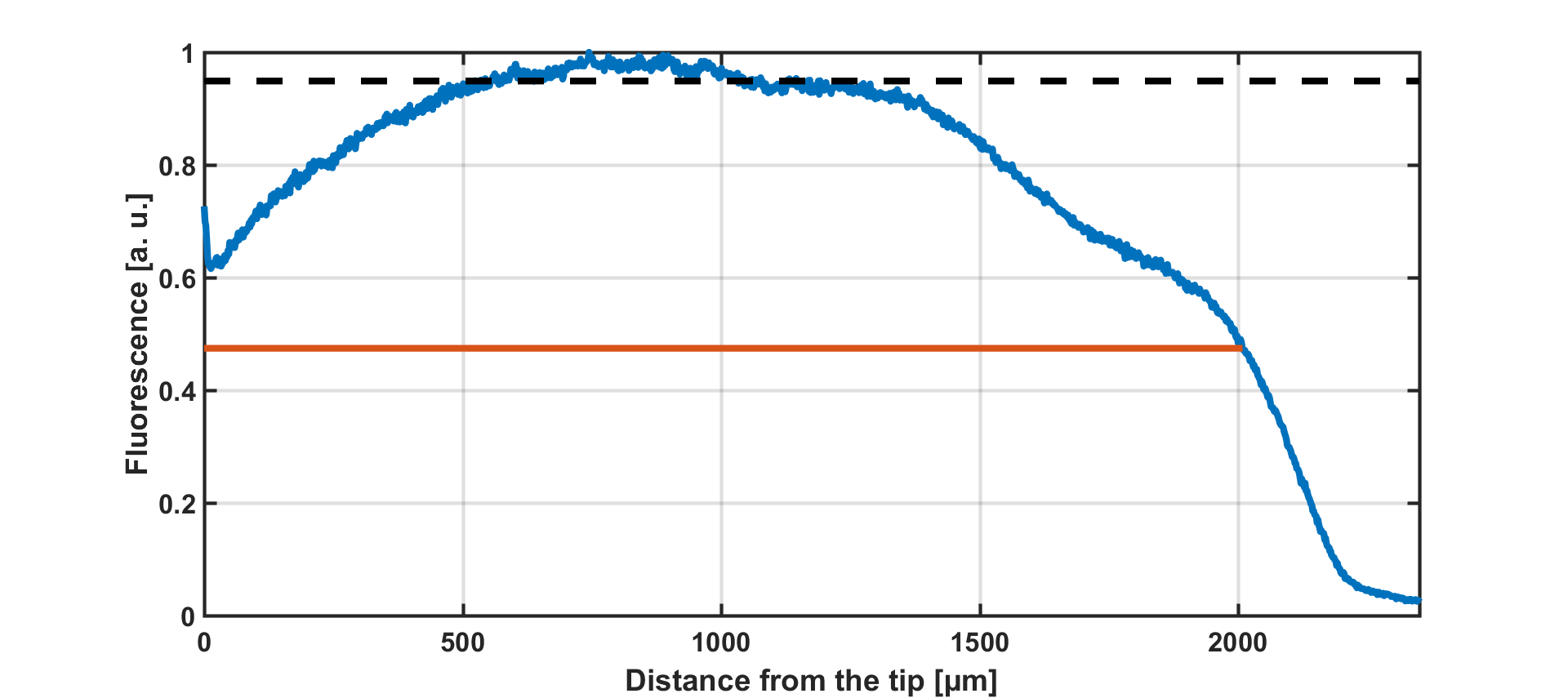


**Supplementary figure 2:** Definition of the First Emission Diameter as the waveguide diameter at

the taper section at which light intensity recorded by the sCMOS sensor (blue curve) fall below half of the value indicated by the black dashed line, representing the average intensity of the data points exceeding 90% of the maximum recorded intensity. The red line highlight the region between the taper tip and the FED.

**Supplementary figure 3**


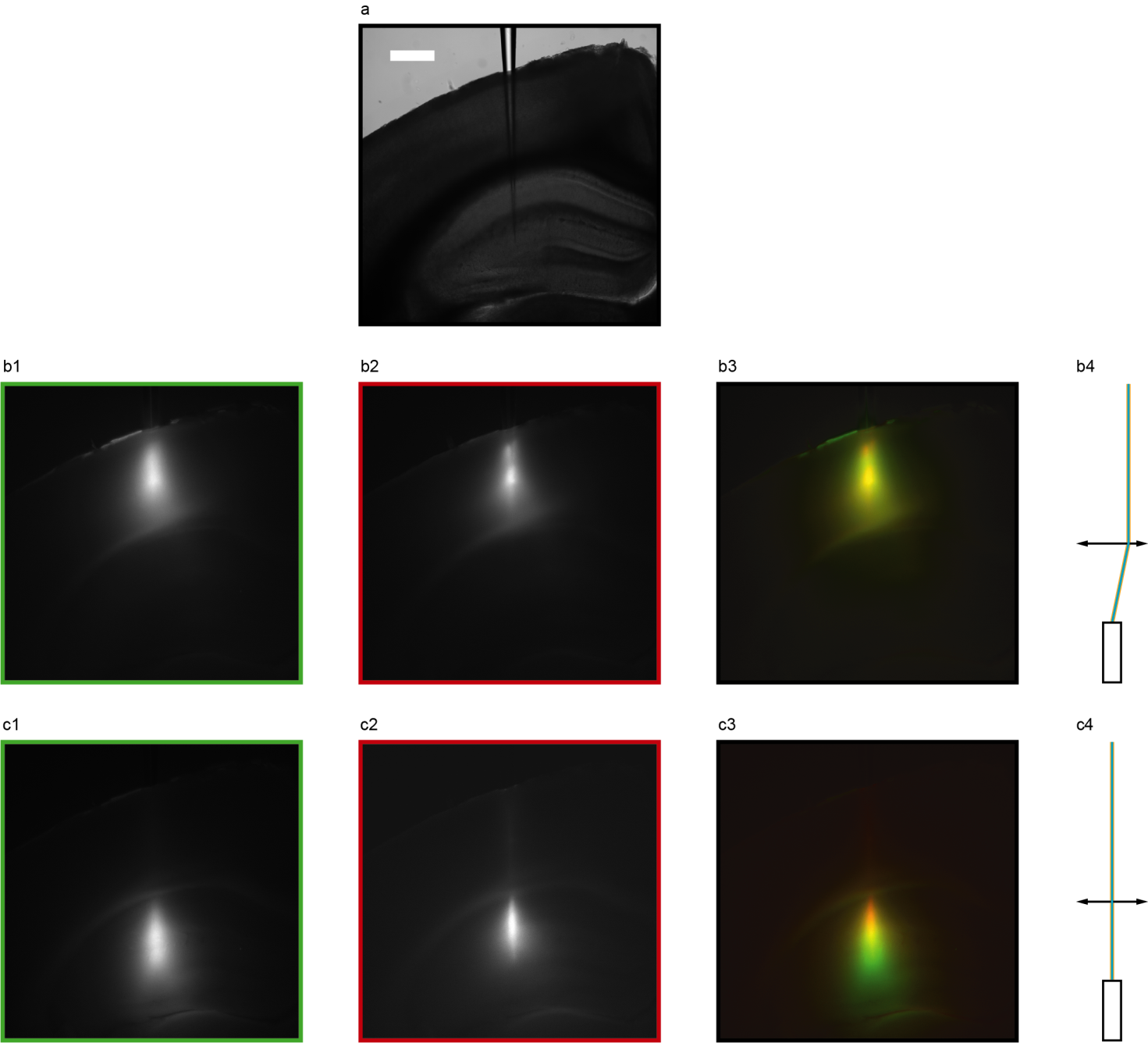


**Supplementary figure 3**: (a) Brightfield image of the location of a NA=0.66 TF inserted in a mouse coronal brain slice with the tip reaching the hippocampus. Scale bar is 500µm and is common to all micrographs in the figure. (b) Green (b1), red (b2) and overlay (b3) channels of simultaneous excitation of SYBR green fluorescence and Mito-Tracker deep-red fluorescence in the cortex. Panel (b4) schematically shows the light injection configuration. (c) Green (c1), red (c2) and overlay (c3) channels of simultaneous excitation of SYBR green fluorescence and Mito-Tracker deep-red fluorescence in the hippocampus. Panel (c4) schematically shows the light injection configuration.

**Supplementary figure 4**

**
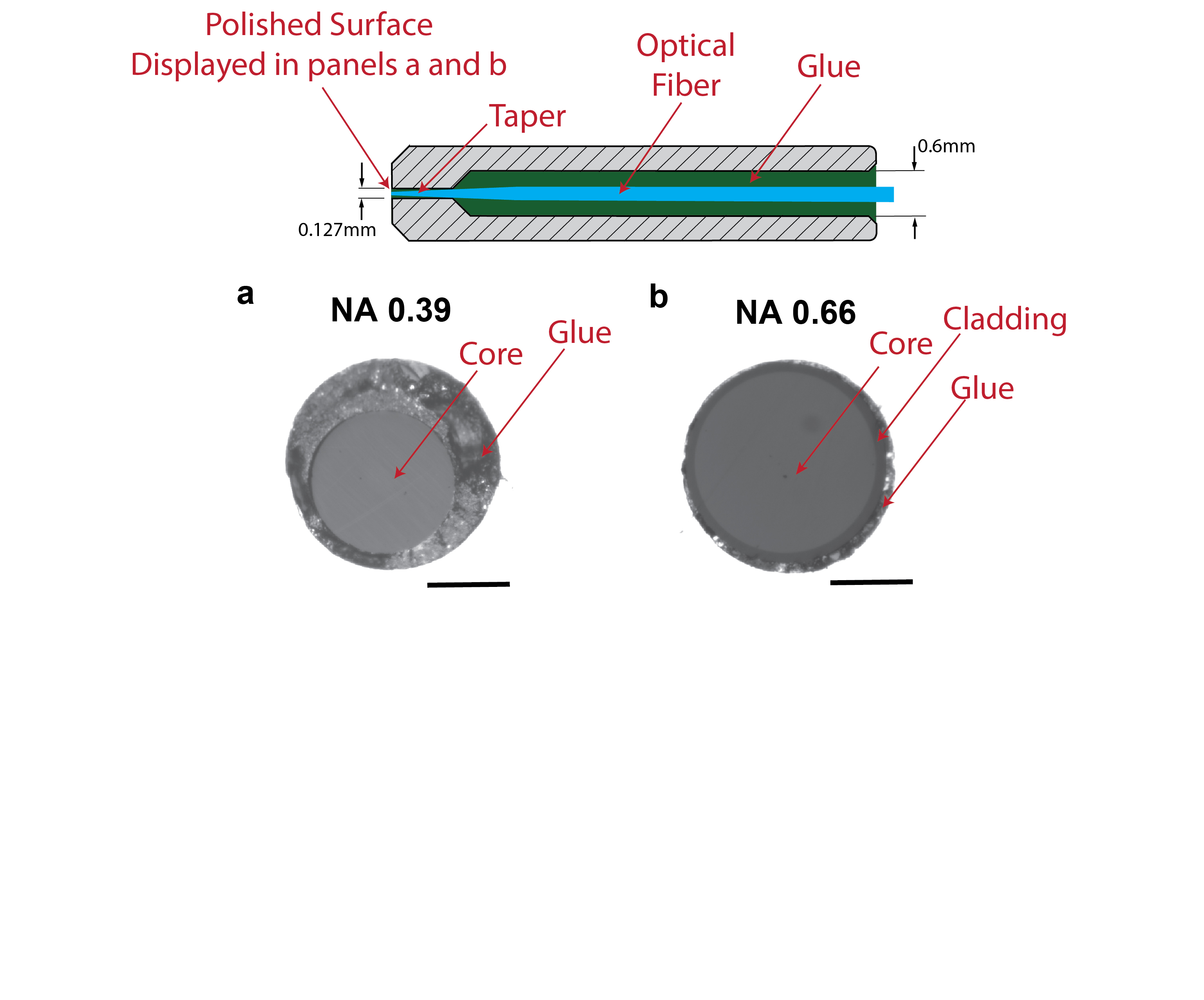
**

**Supplementary figure 4:** Cross section images of the tapered section of NA=0.39 fibers (panel a) and NA=0.66 fibers (panel b), showing the different core/cladding configuration along the taper. Panel b shows a residual cladding not visible in panel (a). Scale bars are 50 µm. The top panel shows how the tapers were mounted in order to take the images in panels (a) and (b). The tapers were inserted in a 127 µm bore ferrule and polished. The TFs underfill the ferrule openings.

**Supplementary figure 5**

**
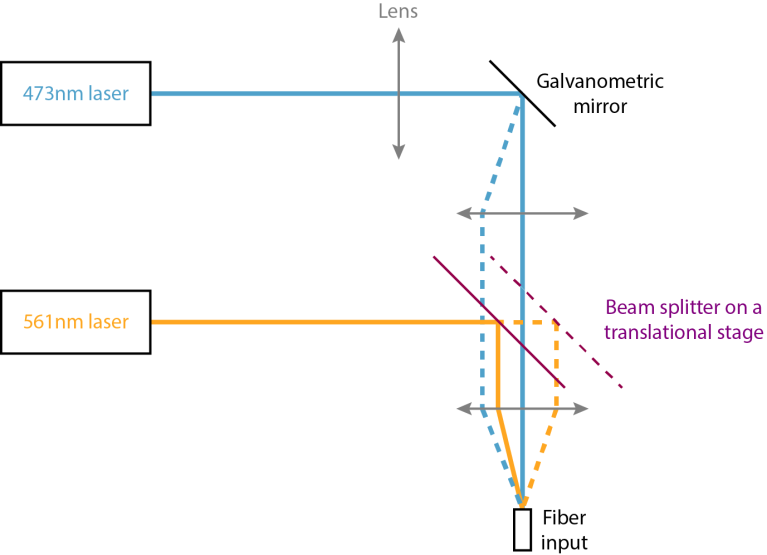
**

**Supplementary figure 5:** Schematic representation of an optical setup allowing the simultaneous injection of two wavelengths in the fiber. The two laser beams are joined through a beam splitter placed on a translational stage before the lens coupling light to the fiber core, acting also as a spatial scanner for the 561nm beam.

**Supplementary figure 6**

**
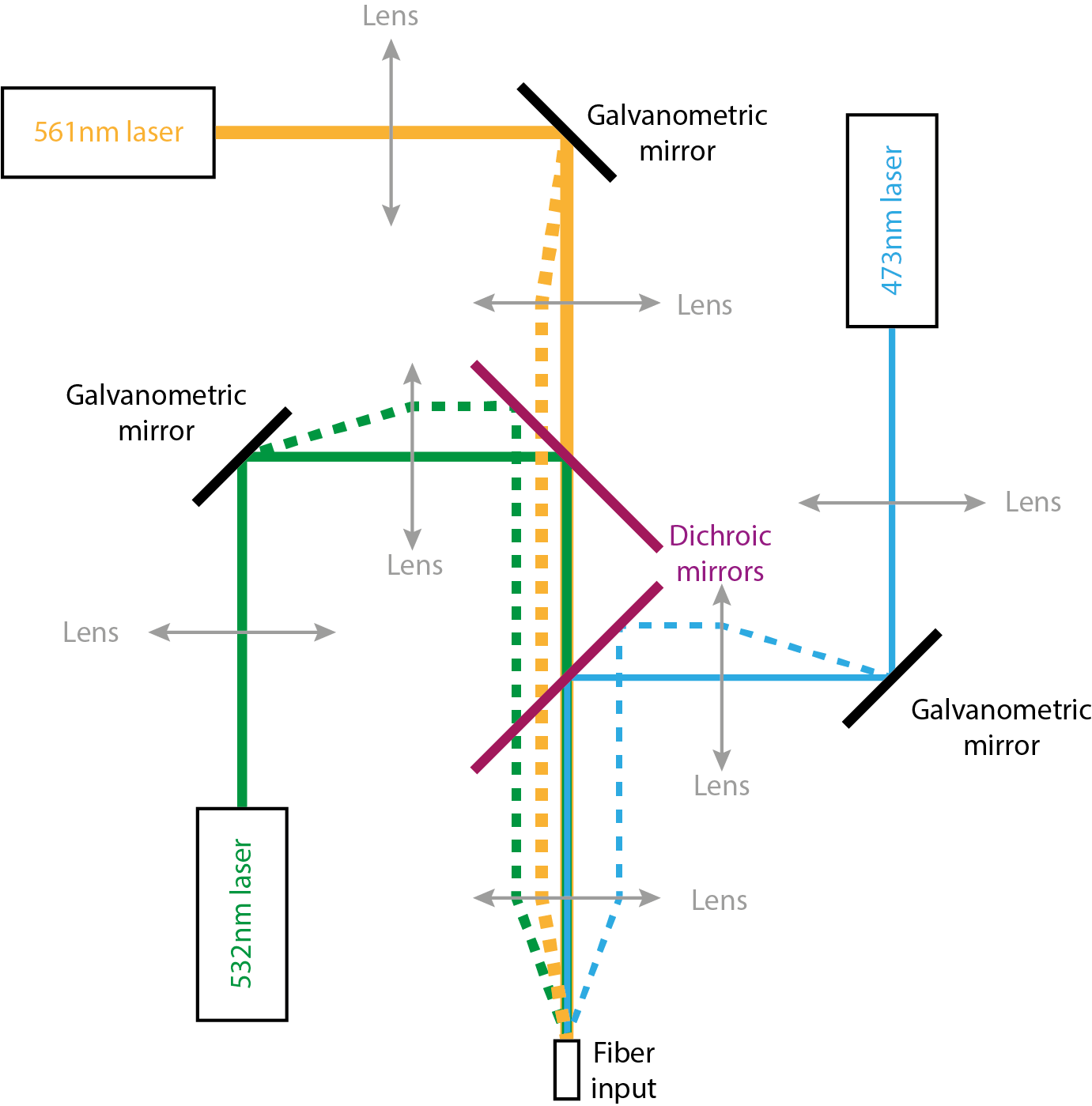
**

**Supplementary figure 6:** Schematic representation of a proposed optical setup to simultaneously inject three wavelengths in the fiber. Each laser beam is deflected by a galvanometric mirror; the three beams are combined through dichroic mirrors and coupled into the fiber by a single achromatic and aspheric lens.
